# “Gol-Gappa” Dystonia – A Novel Form of Focal Task-Specific Dystonia

**DOI:** 10.5334/tohm.1080

**Published:** 2025-10-14

**Authors:** Ayush Thakkar, Narendrakumar Barad

**Affiliations:** 1 Neuroscience Department, Marengo CIMS Hospital, Ahmedabad, Neurology Residency Applicant, India; 2 Marengo CIMS Hospital, Ahmedabad, Gujarat, 380052, India

**Keywords:** Gol-Gappa dystonia, task-specific dystonia, occupational dystonia, writer’s cramp, botulinum injection

## Abstract

**Background:**

Task-specific dystonias (TSDs) are focal movement disorders triggered by highly repetitive, skilled motor tasks, often within profession-specific contexts. Although upper limb TSDs are well documented, culturally unique forms continue to emerge.

**Case Report:**

We present a case of a 30-year-old right-handed male who developed focal dystonia exclusively while preparing Gol-gappa (paani puri), an Indian street food requiring repetitive, precise hand movements to fill puris with spiced stuffing. Neurological examination and neuroimaging were unremarkable. Electromyography guided botulinum toxin injections into affected forearm and hand flexors resulted in approximately 70% improvement in task performance within two weeks.

**Discussion:**

Gol-gappa dystonia represents a novel, culturally specific variant of occupational TSD. This case broadens the recognized spectrum of task-specific dystonias and supports the role of targeted botulinum therapy as an effective intervention.

## Introduction

Task-specific dystonias (TSDs) are focal dystonias of unknown origin, marked by abnormal positioning of specific body parts during particular motor tasks. These tasks frequently require precise and repetitive muscle movements, often associated with high levels of skill [1,2]. For much of the 20th century, task-specific dystonias (TSDs) were mistakenly believed to be psychogenic in nature and were labeled as occupational neuroses. However, in 1982, Sheehy and Marsden identified the true dystonic characteristics of these disorders and demonstrated the absence of any psychological basis in their patients with TSDs [3].The upper extremities and lower facial muscles are frequently affected in various forms of TSDs. Profession-related upper extremity TSDs include shoemaker’s dystonia [2], tailor’s dystonia [2], pianist’s dystonia [4], writer’s cramp [5], as well as cramps in hairdressers [6] and telegraphists [2]. In the context of sports, upper limb TSDs such as pistol shooter’s cramp [7], golfer’s yips [8] weavers’ dystonia [9] and petanque (a French precision ball game similar to bocce) player’s arm dystonia [10] have been identified. More contemporary examples include typing-related dystonia and dystonia observed in esports players [11]. In musicians, TSDs may involve not only pianists but also wind instrumentalists with embouchure dystonia [12], affecting the lips and facial muscles, and string instrumentalists with upper-limb dystonia [4]. Though less common, TSDs can also impact the cervical region and lower limbs. Here, we describe a culturally specific form of TSD related to the gol-gappa profession — Gol-gappa dystonia. Gol-gappa (also known as pani puri or puchka) is a popular Indian street food consisting of crispy, hollow puris filled with a stuffing made from ingredients such as tamarind chutney, boiled potatoes, chickpeas, and various spices.

The preparation of this snack involves repetitive, precise hand movements, particularly in filling the puris with the stuffing, which requires fine motor control. A Gol-gappa business typically entails establishing a food stall or shop where these snacks are freshly prepared and served, often in high-demand environments with continuous customer interaction. In addition, we also want to highlight that the patient has almost 70% improvement in his task-specific functionality after electromyography-guided local botulinum injection in the involved muscles.

## Case report

A 30-year-old right-handed male, employed in the preparation and sale of Gol-Gappa (a popular Indian street food) for 12 years, presented with a 5-year history of insidious-onset, progressive abnormal posturing of the right hand and index finger during task-specific movements. Symptoms exacerbated over the past two years, particularly during evening hours with increased customer demand. The patient denied prior trauma, neuroleptic exposure, or family history of consanguinity, dystonia, or other neurological disorders. Birth and developmental histories were unremarkable.

During his first consultation, the clinical, neurological, and other system examinations were unremarkable. He came with a video showing himself making Gol gappa at his local roadside shop with difficulty he is facing during the same. The video showed – abnormal right wrist flexion, abnormal right index finger flexion at the proximal interphalangeal joint and distal interphalangeal joint, abnormal flexion at the distal interphalangeal joint of the thumb, loss of dexterity, and slowness in executing the particular movement of filling the stuffing (commonly referred to as ‘masala’) in the ‘puri’ (Video-1). Routine hematological, biochemical (renal/liver function, electrolytes, lipid profile, thyroid studies), and metabolic (serum copper, ceruloplasmin) investigations were within normal limits. Genetic testing for DYT1 was considered but not performed taking into account the financial constraints of the patient and absence of family history. Neuroimaging (brain MRI with susceptibility-weighted imaging [SWI], CT scan of the brain) revealed no structural abnormalities. After a detailed discussion of treatment options, the patient received local intramuscular injections of onabotulinumtoxin A under electromyographic (EMG) guidance. The following muscles were targeted:

1. Flexor digitorum profundus – 10 units to the index finger and 5 units to the middle finger muscle belly
2. Flexor digitorum superficialis – 15 units
3. Flexor carpi ulnaris – 10 units
4. Flexor carpi radialis – 10 units
5. Flexor pollicis longus – 10 units

**Video 1:** Abnormal right wrist flexion, right index finger flexion at the proximal interphalangeal joint and distal interphalangeal joint, and flexion at the distal interphalangeal joint of the thumb, loss of dexterity, and slowness in executing the particular movement of filling/pouring masala in Gol-gappa.

After 15 days, he found almost 70% improvement in his problem of Gol gappa making (Video-2). Although some weakness is commonly reported by patients after onabotulinum A toxin injection, this patient did not report any weakness or other side effects.

**Video 2:** Recorded after 15 days of botulinum injection showing significant improvement of the above-mentioned dystonic posturing and increased speed of executing the particular movement of filling/pouring masala in Gol-gappa.

## Discussion

Task-specific Dystonias (TSDs) represent a subset of isolated focal dystonias characterized by their distinctive manifestation during the execution of highly skilled, overlearned motor tasks, with initial symptoms typically confined to the triggering activity [13,14]. Emerging pathophysiological models propose a multifactorial etiology involving network-level dysfunction across cortico-striatal-thalamocortical and cerebellar-thalamic circuits, marked by— **1**. Loss of inhibitory control within sensorimotor pathways, leading to simultaneous contraction of antagonistic muscle groups **2**. Maladaptive neuroplasticity, driven by repetitive task performance and impaired homeostatic regulation of synaptic excitability [2] and/or **3**. Cerebellar contributions where aberrant afferent signaling disrupts basal ganglia integration of sensorimotor feedback, exacerbating motor program dysregulation [15].

Recent evidence highlights the cerebellum’s role in modulating basal ganglia activity through disrupted dentato-thalamic projections, creating aberrant sensorimotor loops that perpetuate task-specific motor discoordination. This mechanism complements traditional models of basal ganglia-centric pathophysiology, emphasizing the interplay between environmental triggers (e.g., repetitive movements) and intrinsic neural vulnerabilities [2,14].

The risk factors for task-specific dystonias (TSDs) remain incompletely defined, though several are consistently reported. Male sex and genetic predisposition (with up to one-quarter of patients reporting a family history) are recognized contributors. The average age of onset typically falls between third and sixth decade of life. TSDs are also associated with high-intensity, long-duration repetition of finely tuned tasks, and they show a predilection for body regions with large cortical representation in the homunculus, particularly the hand and orofacial muscles [15]. Although this long-standing notion has been challenged by more recent evidence that suggests that neural representations in primary sensory and motor cortices rather remain intact and the dysfunction may emerge in higher-order skill encoding [16]. Certain psychological traits, such as perfectionism or heightened performance pressure, have also been implicated as potential modulators [13].

Our patient embodies several of these risk factors: he is male, developed symptoms in his early thirties, and had practiced a highly repetitive, skilled motor task for over a decade involving the hand. Interestingly, his symptoms were most pronounced during high-demand customer hours, suggesting that environmental factors such as increased task intensity and situational stress may exacerbate symptom severity.

Recent motor-control models and reviews suggest that task features strongly influence the risk of developing task-specific dystonia (TSD): tasks that incorporate greater movement variability, lower demands for sub-second precision, or lower/intensely variable repetition are less likely to drive the maladaptive sensorimotor plasticity implicated in TSD [13,15]. Conversely, skills characterized by extremely high repetition, little variability, and the need for rapid, highly fractionated movements — especially when practiced intensively — confer greater risk, as repeatedly documented in musician cohorts [13,17]. Tasks that require movement patterns that depart substantially from a body part’s natural repertoire also appear to increase vulnerability, presumably by forcing atypical sensorimotor mappings that encourage maladaptive reorganization [2,18]. Finally, individual vulnerability (including genetic predisposition and abnormalities of inhibitory cortical and subcortical networks) interacts with these task features to determine whether and how the clinical phenotype emerges [2,15].

Our patient developed a fascinating and highly specific form of dystonia, triggered only during Gol-gappa making, a task he had practiced meticulously for years. This activity requires a unique combination of rapid, repetitive pinch-and-fill movements performed under time pressure. The biomechanics of this action make the dystonia novel compared with more classically described tasks such as writing or playing a string instrument. His wrist flexed involuntarily and his fingers clenched into an abnormal posture, yet he could still button his shirt, eat, and perform other daily tasks without difficulty. This striking task-selective phenomenon places his condition within the spectrum of task-specific dystonias (TSDs) and illustrates how the brain’s motor control can falter under precise, repetitive demands. TSD has been reported in many professions, but to our knowledge never in Gol-gappa making—making this a novel addition to the occupational TSD literature.

The dosing strategy for onabotulinum toxin A in our patient was guided by prior reports of focal hand and task-specific dystonias and current consensus recommendations. Typical per-muscle doses for small forearm/hand muscles (e.g., flexor digitorum profundus, flexor digitorum superficialis, flexor carpi ulnaris, flexor carpi radialis, flexor pollicis longus) are commonly in the range of approximately **5–20 units per muscle**, individualized according to EMG activation and clinical severity; cumulative session doses for occupational TSDs such as writer’s cramp or musician’s dystonia are often in the **~50–100 U** range (with substantial case-by-case variation). We therefore used a total dose of **60 U**, tailored to EMG findings and clinical response, and observed ~70% functional improvement without clinically meaningful weakness, supporting the appropriateness of our regimen in this case [19,20,21].
